# Inverse probability weighting to estimate causal effect of a singular phase in a multiphase randomized clinical trial for multiple myeloma

**DOI:** 10.1186/s12874-016-0253-9

**Published:** 2016-11-09

**Authors:** Annalisa Pezzi, Michele Cavo, Annibale Biggeri, Elena Zamagni, Oriana Nanni

**Affiliations:** 1 Istituto di Ematologia Seràgnoli, Università degli Studi di Bologna, Policlinico Sant’Orsola-Malpighi, Via Massarenti 9, 40138 Bologna, BO Italy; 2 Dipartimento di Statistica, Informatica, Applicazioni “Giuseppe Parenti” (DiSIA), Università di Firenze, Via Morgagni 57-59, 50134 Firenze, FI Italy; 3 SC Biostatistica, Istituto per lo studio e la prevenzione oncologica (ISPO), Via delle Oblate 2, 50126 Firenze, FI Italy; 4 Istituto Scientifico Romagnolo per lo Studio e la Cura dei Tumori (IRST) IRCCS, Via Maroncelli 40, 47014 Meldola, FC Italy

**Keywords:** Selection bias, Weighting sample, Propensity score, Causal effect, RCT, Compliance

## Abstract

**Background:**

Randomization procedure in randomized controlled trials (RCTs) permits an unbiased estimation of causal effects. However, in clinical practice, differential compliance between arms may cause a strong violation of randomization balance and biased treatment effect among those who comply. We evaluated the effect of the consolidation phase on disease-free survival of patients with multiple myeloma in an RCT designed for another purpose, adjusting for potential selection bias due to different compliance to previous treatment phases.

**Methods:**

We computed two propensity scores (PS) to model two different selection processes: the first to undergo autologous stem cell transplantation, the second to begin consolidation therapy. Combined stabilized inverse probability treatment weights were then introduced in the Cox model to estimate the causal effect of consolidation therapy miming an ad hoc RCT protocol.

**Results:**

We found that the effect of consolidation therapy was restricted to the first 18 months of the phase (HR: 0.40, robust 95 % CI: 0.17-0.96), after which it disappeared.

**Conclusions:**

PS-based methods could be a complementary approach within an RCT context to evaluate the effect of the last phase of a complex therapeutic strategy, adjusting for potential selection bias caused by different compliance to the previous phases of the therapeutic scheme, in order to simulate an ad hoc randomization procedure.

**Trial registration:**

ClinicalTrials.gov: NCT01134484 May 28, 2010 (retrospectively registered)

EudraCT: 2005-003723-39 December 17, 2008 (retrospectively registered)

## Background

Randomized controlled trials (RCTs) are the gold standard for clinical research because the randomization procedure is expected to achieve a balanced distribution of both known and unknown baseline characteristics between experimental and control treatment arms [1]. Unfortunately, selection mechanisms may occur after randomization, i.e. some patients are lost to follow up or drop out early or occurrence of competing risk events such as death from other diseases. Reasons for early drop out include informed consent withdrawal or toxicities. When a large number of patients do not comply with the therapeutic protocol, the effect of treatment may be difficult or impossible to estimate. In fact, the consequence of selection bias is that the association between treatment and outcome among those selected for analysis (because entirely compliant with treatment protocol) may differ from that among those who are eligible. Patients may decide not to follow, partially follow, or entirely follow a complex therapeutic program for reasons related to the outcome [2]. The intention-to-treat (ITT) analysis provides an unbiased estimate of gross treatment effect but is shrunk toward the null value on the basis of the percentage of compliers. Conversely, the per-protocol (PP) analysis may introduce bias because the groups of patients being compared no longer have similar characteristics. Thus, it is possible that some patients are more likely to be treated than others (possibly depending on prognostic factors) in which case treatment may erroneously appear more efficacious [3–5].

Another important issue is that RCTs usually compare the entire therapeutic strategy without any detailed evaluation of each singular therapeutic phase. The evaluation of a specific phase is complicated because it may be biased by different compliance to the previous phases. In fact, during the course of a trial, especially when the study protocol is complex and the disease is severe, randomization balance may be lost right from the earliest phases due to selective patient withdrawal between one phase and another.

Several methods have been proposed to tackle these problems in an RCT setting where post-randomization imbalance occurs, and also within the context of observational studies where randomization is not possible. One popular approach is the use of the propensity score (PS), a method developed to reduce the effect of observable confounding factors [6].

The aim of this article was to evaluate the effect of the consolidation therapy, the last phase of a complex therapeutic strategy composed by two arms of induction therapy randomly administered to prepare patients to receive at least one autologous stem cell transplantation (ASCT), in patients with previously untreated multiple myeloma. Only patients that received at least one ASCT can proceed to receive consolidation therapy. Those receiving consolidation therapy receive the same therapy to which they were randomized during the induction phase. For these reasons, we needed to adjust for potential selection bias due to different compliance to previous phases of therapeutic protocol in order to approximate an RCT with randomization procedure (consolidation vs not consolidation phase) after ASCT (with time 0 being the end of the last ASCT received). We did it by modelling both the probability to receive at least 1 ASCT and the probability to receive consolidation phase through a combined PS approach.

This study is justified by the increasing interest being shown in consolidation therapy on progression free survival endpoint [7]. Consolidation treatment is one of the latest phases of MM therapeutic protocols and aims to further increase the frequency and depth of clinical response obtained with previous treatments phases.

The paper is structured as follows. In section 2, we describe the statistical methods used, in particular, the definition of the PS, the weighting procedure in the survival analysis, and the Aalen’s additive hazards model to investigate time-varying covariates. In section 3, we report the results of the role of the consolidation phase in the treatment of MM. In section 4 and 5, we discuss the results and draw our conclusions.

## Methods

In this section we describe the statistical methods used to restore randomization balance to obtain an unbiased estimate of the causal effect of a treatment. In particular, we review the definition of the PS and its possible uses, the weighting procedure in survival analysis, and the Aalen’s additive hazards model to investigate time-varying effects.

### Propensity score

The propensity score (PS) is the conditional probability of being treated given a set of observed potential confounders. In this way all the information from a large number of potential confounders is summarized into a unique balancing score variable (the so-called *propensity score*). The PS may warrant that the distribution of measured baseline covariates is the same in treated and untreated subjects. Rosenbaum and Rubin said that PS can account for imbalance in treatment groups and reduce bias by simulating a sort of “virtual randomization” of subjects into treatment groups (conditional exchangeability) [6].

The PS is the conditional probability of receiving a treatment given pretreatment characteristics:

$$ \mathrm{p}\left(\mathbf{X}\right)= \Pr \left(\mathrm{Z}=1\Big|\mathbf{X}\right) $$

where Z = {0, 1} is the indicator of exposure to treatment and **X** is the multidimensional vector of pretreatment characteristics. Thus, if exposure to treatment is random within cells defined by **X**, it is also random within cells defined by the values of the one-dimensional variable p(**X**). Bias-removing adjustments can therefore be made using the PS alone rather than modeling all of the background covariates individually.

The expected difference in observed responses at p(**X**) is equal to the average treatment effect at p(**X**), E{r_1_|p(**X**), Z = 1} - E{r_0_|p(**X**), Z = 0} = E{r_1_ - r_0_|p(**X**)} where *r* = {0, 1} is the indicator of the response that would have resulted if a patient had or had not received treatment, respectively; these are the potential outcomes in the two counterfactual situations of treatment and no treatment ([6], Theorem 4 in Rosenbaum and Rubin); [8, 9].

There is still some debate about how to determine a sufficient set of covariates in the PS model. Brookhart states that variables that are unrelated to the exposure but related to the outcome should always be included in a PS model. The inclusion of these variables decreases the variance of an estimated treatment effect without increasing bias. In contrast, including variables that are related to the exposure but not to the outcome increases the variance of the estimated exposure effect without decreasing bias [10]. Of course if a variable is related to both exposure and outcome, it must be included. Conversely, Pearl argues that, although treated and untreated units are balanced in each stratum of the PS, the balance only holds relative to the covariates measured; unobserved confounders may be highly unbalanced in each stratum. “Such imbalance may be dormant in the crude estimate and awakened through the use of PS methods” ([11], page 1415). The effectiveness of PS methods rests critically on the choice of a sufficient set of covariates, which requires in depth knowledge about the causal relationships among both observed and unobserved covariates [12].

### Inverse probability of treatment weight

Austin explains that there are mainly four ways of using the PS to reduce or minimize the effects of confounding when estimating the effects of treatments on outcomes: matching on the PS, stratification on the PS, inverse probability of treatment weighting (IPTW) using the PS, and covariate adjustment using the PS [13]. We chose the IPTW method because we were mainly interested in population effect, i.e. the average treatment effect over the marginal distribution of observed covariates in the study sample. Moreover, Austin’s results showed that, while all ways of using PS allow for the estimation of marginal hazard ratios with minimal bias, IPTW provides estimates with lower mean squared error; it is also less subject to loss of information due to lack of matching.

IPTW is calculated by the inverse of the conditional probability of receiving the exposure that a patient indeed received:

$$ \mathrm{w}=\frac{\mathrm{Z}}{\mathrm{p}\left(\mathbf{\mathsf{X}}\right)}+\frac{\left(1-\mathrm{Z}\right)}{1-\mathrm{p}\left(\mathbf{\mathsf{X}}\right)} $$

where Z indicates whether or not the subject was treated while p(**X**) represents the conditional probability for the subject to be treated. Both treated subjects with a very low PS and untreated subjects with a high PS have large IPTWs to account for unequal probability of receiving treatment in the original sample.

The aim of IPTW is to reduce selection bias by creating a “pseudo-population” in which the exposure is independent of the measured confounders so that the treatment effect estimate in a sample thus weighted will be less biased [14–17].

### Stabilized inverse probability of treatment weight

It may happen that treated subjects have a PS near 0 or that untreated subjects have a PS near 1, making the relative IPTW excessively high and unstable. Computationally, Xu and Ross noticed that, as in any weighted regression, unstabilized IPTW changes the sample size of the original sample, generating an underestimate of the variance of the estimate of the effect, producing inappropriately narrow confidence intervals and leading to the lack of control of the probability of a type I error [15].

Stabilized inverse probability of treatment weight (SIPTW) can be obtained by multiplying the IPTW by the marginal probability of receiving the actual treatment received. Moreover, it preserves the sample size of the original data, produces appropriate estimation of the variance of the main effect, and adequately controls the type I error rate [18, 19].

### Adjusted Kaplan-Meier survival curves

Cole et al. demonstrated that, under the assumption of no unmeasured confounding, adjusted Kaplan-Meier estimates for survival curves of treated and untreated - where each subject is weighted by its IPTW - represent the survival curve of the entire sample had none been exposed, and the survival curve of the entire sample had everyone been exposed, respectively [19]. Xie et al. showed that the adjusted Kaplan-Meier estimator is consistent even if weights came from the PS computed by a logistic regression model [20]. This is also true for stabilized inverse probability weighted estimates [21].

### Adjusted Cox proportional hazard model

Cole et al. demonstrated that the stabilized inverse probability of treatment weighting (SIPTW) Cox regression model provides unbiased estimates, while robust variance estimation, such as those suggested by Lin and Wei, can be used to account for the weighting procedure. These results are also valid in the presence of time-varying confounding [18, 19, 22].

### Aalen’s additive hazards model

Exploration of SIPTW weighted Kaplan-Meier survival curves is recommended in the presence of violation of the proportional hazard (PH) assumption [21]. In this case, Aalen’s additive hazards model represents a valid exploratory graphical method to detect and describe the nature of time-varying covariate effects [23]. The hazard function at time t for a model containing *p+1* covariates is: h{t,**X**,β(t)} = β_0_(t) + β_1_(t)x_1_ + β_2_(t)*x*_2_ + … β_p_(t)x_p_.

The coefficient β_k_(t) provides the change in hazard at time t from the baseline hazard function β_0_(t) for a one-unit change in the respective covariate x_k_, holding all other covariates constant. A weighted version of the Aalen’s model was discussed by Huffer and McKeague [24].

The principal difference between Aalen’s and Cox models is that Aalen’s model allows the effect of the k^th^ covariate to change continuously over time and represents a valid method to describe the function. The Aalen estimator is based on the cumulative hazard function (obtained by integrating the hazard function at time t for a model containing *p+1* covariates) where β_k_(t) represents the cumulative regression coefficient for the k^th^ covariate over time. A plot of ${\displaystyle {\widehat{\beta}}_k}(t)$ versus t with its 95 % confidence interval is a worthwhile method to identify and describe the possible interaction between the coefficient with time and also to evaluate its relevance.

Once a significant time-varying effect has been found for a given covariate, a simple approach would be to introduce it into the Cox proportional hazard model as a time-varying covariate and to estimate a specific hazard ratio for each particular time-interval identified. An appropriate modeling of the time-varying covariates makes the Cox model suitable for satisfying the proportional hazard assumption.

## Results

Study population characteristics and results of the role of the consolidation phase in the treatment of MM are described in this section.

### Study design

The original study consisted of 480 patients enrolled between May 2006 and April 2008 from 73 Italian hospitals in a phase III open-label randomized clinical trial (RCT). Eligible patients were aged 18–65 years and had previously untreated symptomatic and measurable multiple myeloma [25].

Enrolled patients were randomized (1:1 ratio) into two treatment arms: the experimental arm (Arm A) versus the standard arm (Arm B). Six patients withdrew consent immediately after randomization without starting therapy. In both arms, patients received induction therapy before undergoing up to two planned autologous stem cell transplantation (ASCT) procedures followed by a consolidation phase. The experimental/standard therapy was administered in both the induction and consolidation phases.

The study was approved by an independent ethics committee or by the institutional review board of each participating institution, and was performed in accordance with International Conference on Harmonisation guidelines on Good Clinical Practice and with the principles laid down in the Declaration of Helsinki. All patients provided written informed consent prior to enrollment.

Complete information on pre-treatment baseline characteristics was available for 414 (87.34 %) of the 474 randomized patients (Table 1), which represents the core of the analysis.

**Table 1:** Baseline characteristics according to consolidation compliance

Characteristic		Baseline characteristics	Consolidation treatment		
		414	None received 118 (28.50 %)	Received 296 (71.50 %)	P
Age (years)		Mean (SD)	Mean (SD)	Mean (SD)	
		56.00 (7.21)	57.08 (6.48)	55.57 (7.44)	0.07
		Median (IQR)	Median (IQR)	Median (IQR)	
		57.47 (51.57–61.74)	58.23 (52.40–62.28)	56.76 (51.08–61.49)	
Sex	Male	236 (57.00 %)	62 (52.54 %)	174 (58.78 %)	0.25
	Female	178 (43.00 %)	56 (47.46 %)	122 (41.22 %)	
Haemoglobin (g/dL)	>10.5 normal	251 (60.63 %)	67 (56.78 %)	184 (62.16 %)	0.31
	≤10.5 abnormal	163 (39.37 %)	51 (43.22 %)	112 (37.84 %)	
Platelets (×10^9^ per L)	>150 normal	369 (89.13 %)	107 (90.68 %)	262 (88.51 %)	0.52
	≤150 abnormal	45 (10.87 %)	11 (9.32 %)	34 (11.49 %)	
Creatinine (μmol/dL)	≤1.2 normal	326 (78.74 %)	94 (79.66 %)	232 (78.38 %)	0.77
	>1.2 abnormal	88 (21.26 %)	24 (20.34 %)	64 (21.62 %)	
LDH (U/L)	≤190 normal	59 (14.25 %)	24 (20.34 %)	35 (11.82 %)	0.03
	>190 abnormal	355 (85.75 %)	94 (79.66 %)	261 (88.18 %)	
ISS stage	1	180 (43.48 %)	44 (37.29 %)	136 (45.95 %)	0.26
	2	162 (39.13 %)	50 (42.37 %)	112 (37.84 %)	
	3	72 (17.39 %)	24 (20.34 %)	48 (16.22 %)	
IgA isotype	IgA	81 (19.57 %)	25 (21.19 %)	56 (18.92 %)	0.60
	Not IgA	333 (80.43 %)	93 (78.81 %)	240 (81.08 %)	
Del(13q)	absent	214 (51.69 %)	61 (51.69 %)	153 (51.69 %)	0.99
	present	200 (48.31 %)	57 (48.31 %)	143 (48.31 %)	
Del(17p)	absent	385 (93.00 %)	110 (93.22 %)	275 (92.91 %)	0.91
	present	29 (7.00 %)	8 (6.78 %)	21 (7.09 %)	
T(4;14)	absent	331 (79.95 %)	98 (83.05 %)	233 (78.72 %)	0.32
	present	83 (20.05 %)	20 (16.95 %)	63 (21.28 %)	
Treatment arm	A experimental	200 (48.31 %)	53 (44.92 %)	147 (49.66 %)	0.38
	B control	214 (51.69 %)	65 (55.08 %)	149 (50.34 %)	

Wilcoxon-Mann-Whitney test was used for continuous variables

Chi square test was used for categorical variables

At a median follow up of 54 months from randomization, 85/200 and 121/214 events/patient (progression, relapse, or death from any cause) had been recorded in the experimental and control arms, respectively.

**Fig. 1:**
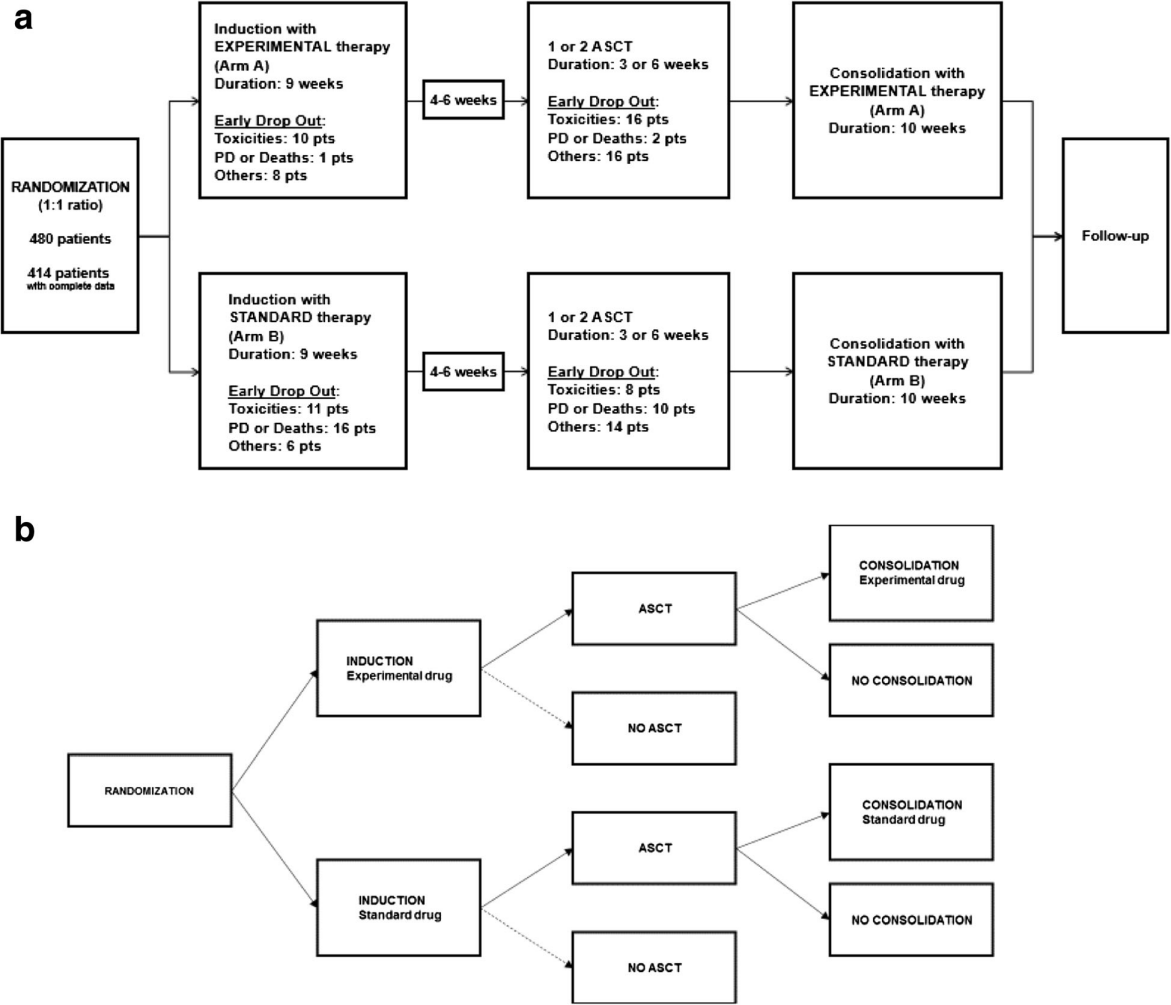
**a** Study design of a phase III open-label RCT carried out in 73 Italian hospitals. Eligible untreated symptomatic multiple myeloma patients aged 18–65 years were randomized (1:1 ratio) to receive experimental (Arm A) versus standard (Arm B) treatment as induction therapy before a maximum of two planned autologous stem cell transplantations (ASCT) followed by a consolidation phase consisting on the same arm of therapy as induction phase. **b** Miming an Ad hoc RCT to evaluate the role of consolidation therapy. Eligible untreated symptomatic multiple myeloma patients aged 18–65 years who had received at least 1 ASCT after having been prepared with induction therapy (Experimental or Standard) were randomized to receive the same arm of therapy (Experimental or Standard) as a consolidation phase or to not receive any therapy

Overall, 52 patients in arm A and 65 in arm B discontinued treatment before undergoing the consolidation phase, mainly because of toxicity in Arm A and disease progression in Arm B (Fig. 1a). To evaluate the specific role of the consolidation phase miming an ad hoc RCT (Fig. 1b) it was therefore necessary to consider the selection process in a differential way on the basis of baseline characteristics, treatment arm and clinical course. In doing so, we were able to restore a balanced comparison for the consolidation phase either conditionally or marginally by weighting for the conditional probability of receiving consolidation therapy.

### Propensity score, inverse probability of treatment weight and stabilized inverse probability of treatment weight estimation

The consolidation phase was administered to patients who received at least one of the two planned ASCT procedures. Two selection processes were then modeled, the first by computing the conditional probability of receiving at least one ASCT and the second by computing the conditional probability of undergoing the consolidation phase.

The first PS – of undergoing at least one of the two planned ASCTs – was calculated on the basis of pre-treatment baseline characteristics (age, sex, hemoglobin, platelets, creatinine, LDH, ISS stage, isotype of disease and cytogenetic abnormalities) and treatment arm. The second PS – of undergoing the consolidation phase – was calculated using pre-treatment baseline characteristics, treatment arm and clinical response to the induction phase (categorized as complete or partial) (Table 2). We showed detailed models because there is still some debate about the choice of the proper covariates in the PS model. To note that, in our case, no variable in the logistic models resulted particularly important. Nevertheless, it may be possible to proceed with the re-weighting procedure.

**Table 2:** Propensity score logistic models of receiving at least one ASCT and of receiving consolidation phase treatment

	Receiving at least one ASCT					Receiving consolidation phase treatment				
	Coeff.	S.E.	P > |z|	95 %	C.I.	Coeff.	S.E.	P > |z|	95 %	C.I.
Age > 50 years	0.2192	0.2005	0.2740	-0.1738	0.6122	-1.1036	0.3268	0.0010	-1.7442	-0.4631
Male sex	-0.3034	0.1756	0.0840	-0.6476	0.0408	0.1046	0.1739	0.5470	-0.2361	0.4454
HB ≤ 10.5 g/dL	-0.1751	0.1809	0.3330	-0.5296	0.1795	0.0556	0.1851	0.7640	-0.3072	0.4183
Plts ≤ 150 x10^9^/L	-0.1085	0.2592	0.6750	-0.6166	0.3995	0.3935	0.3059	0.1980	-0.2060	0.9930
Crea > 1.2 μmol/dL	0.0577	0.2220	0.7950	-0.3774	0.4927	0.0155	0.2167	0.9430	-0.4092	0.4402
LDH > 190 U/L	0.3097	0.2220	0.1630	-0.1254	0.7448	0.3123	0.2309	0.1760	-0.1403	0.7648
ISS > 1	0.2584	0.1864	0.1660	-0.1070	0.6238	0.1231	0.1770	0.4870	-0.2238	0.4700
IgA isotype	0.0031	0.2062	0.9880	-0.4011	0.4073	-0.1299	0.2136	0.5430	-0.5485	0.2887
Del(13q)	0.0375	0.1765	0.8320	-0.3086	0.3835	-0.0420	0.1722	0.8070	-0.3795	0.2954
T(4;14)	-0.1485	0.2099	0.4790	-0.5599	0.2628	0.3715	0.2321	0.1100	-0.0835	0.8264
Del(17p)	-0.2640	0.3039	0.3850	-0.8597	0.3317	0.4939	0.4040	0.2220	-0.2979	1.2858
Arm A	0.2628	0.1672	0.1160	-0.0649	0.5906	0.0036	0.1657	0.9830	-0.3212	0.3284
CR at induction						0.1073	0.2476	0.6650	-0.3781	0.5926
Constant	0.7531	0.2990	0.0120	0.1670	1.3392	1.3012	0.4762	0.0060	0.3678	2.2345

From each PS, the relative inverse probability of treatment weight (IPTW) and the stabilized inverse probability of treatment weight (SIPTW) were calculated as previously described.

Total stabilized inverse probability of treatment weight (TSIPTW) for each patient was obtained by multiplying the SIPTW of undergoing the 1^st^ ASCT by the SIPTW of undergoing the consolidation phase (Table 3). It may be noted that the stabilization procedure made every inverse probability weight more stable than their respective non stabilized weights.

**Table 3:** Estimated inverse probability of treatment weight (IPTW) and stabilized inverse probability of treatment weight (SIPTW) of starting 1^st^ ASCT (A), of starting consolidation therapy (B) and total product (TSIPTW) (C), respectively

	Mean ± Standard Deviation	Range
Starting 1^st^ ASCT (A)		
IPTW	2.00 ± 2.75	1.03–20.88
SIPTW	1.00 ± 0.21	0.29–2.62
Starting Consolidation Therapy (B)		
IPTW	1.87 ± 2.52	1.00–31.75
SIPTW	1.00 ± 0.37	0.43–5.79
Total Product (C)		
IPTW	2.99 ± 3.74	1.09–34.67
SIPTW	1.00 ± 0.42	0.29–5.53

Three hundred and sixty-two (87.44 %) patients started the 1^st^ ASCT with a median PS (PS1) of 0.89 (range: 0.56–0.97; interquartile range (IQR): 0.84–0.93). The SIPTW of starting the 1^st^ ASCT was obtained as **[**0.8744/PS1**]** or **[**(1-0.8744)/(1- PS1)**]** in the event of not starting it. The median value was 0.97 (range: 0.29–2.62; IQR: 0.93–1.03).

Of the 362 who started the 1^st^ ASCT, 81.77 % also started the consolidation phase with a median PS (PS2) of 0.80 (range: 0.58–1.00; IQR: 0.75–0.88).

The SIPTW of starting consolidation therapy was obtained as **[**0.8177/PS2**]** or **[**(1-0.8177)/(1-PS2)**]** in the event of not starting it. The median value was 1.00 (range: 0.43–5.79; IQR: 0.87–1.06).

Finally, the TSIPTW was obtained as the product of SIPTW of starting the 1^st^ ASCT by the SIPTW of starting consolidation therapy. The median value was 0.96 (range: 0.29–5.53; IQR: 0.86–1.07). A TSIPTW > 2 was observed in a minority of patients (*n* = 8) who had not started consolidation therapy. Five of these had not finished induction therapy due to serious toxicities (*n* = 3), progression (*n* = 1) or death (*n* = 1) and were removed from the study; the remaining 3, who had not reached complete clinical response (CR), dropped out of the study after the ASCT because of severe toxicity, progression or protocol violation. For those who did not start the 1^st^ ASCT (*n* = 52), the TSIPTW corresponded to the SIPTW of undergoing the 1^st^ ASCT because the SIPTW of starting consolidation therapy could not be computed, obviously.

### Weighted Kaplan-Meier curves

After having estimated the TSIPTW to undergo the consolidation phase, 346 patients who could potentially receive consolidation therapy as they had undergone at least one ASCT were evaluated in terms of progression-free survival (PFS), defined as the time elapsed from the last ASCT evaluation to the date of progression or death (events), or last follow up (censored).

**Fig. 2:**
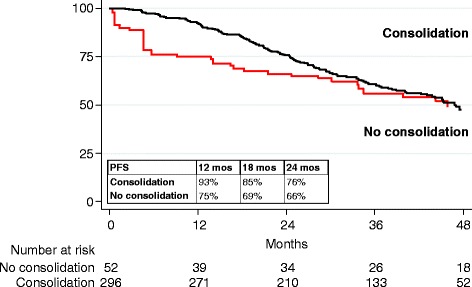
Weighted Kaplan-Meier survival estimates for PFS from last ASCT evaluation date, by consolidation treatment

The consolidation phase did not have an effect on PFS: a gradual decrease was seen in the survival curves of patients who had undergone consolidation therapy, while those who had not started this last phase of therapy rapidly relapsed till about 12 months from the last ASCT evaluation. After the first year, the two PFS survival curves became similar (after TSIPTW 1-year PFS: 93 % vs 75 %, weighted Log-rank test *p* = 0.9554) (Fig. 2). This happened because TSIPTWs gave more weight to patients who did not receive consolidation therapy and who were more likely to fail during the first year after ASCT.

### Aalen’s additive hazards model to investigate time-varying effect

As the PH assumption did not hold (Schoenfeld’s test, *p* < 0.0001), Aalen’s additive hazards models were fit to investigate the time-varying effect of the consolidation phase. We fitted a model containing all the prognostic factors – i.e. age, sex, hemoglobin, platelets, creatinine, ISS stage, cytogenetic alterations – and treatment arm and an indicator for the consolidation phase (yes/no).

**Fig. 3:**
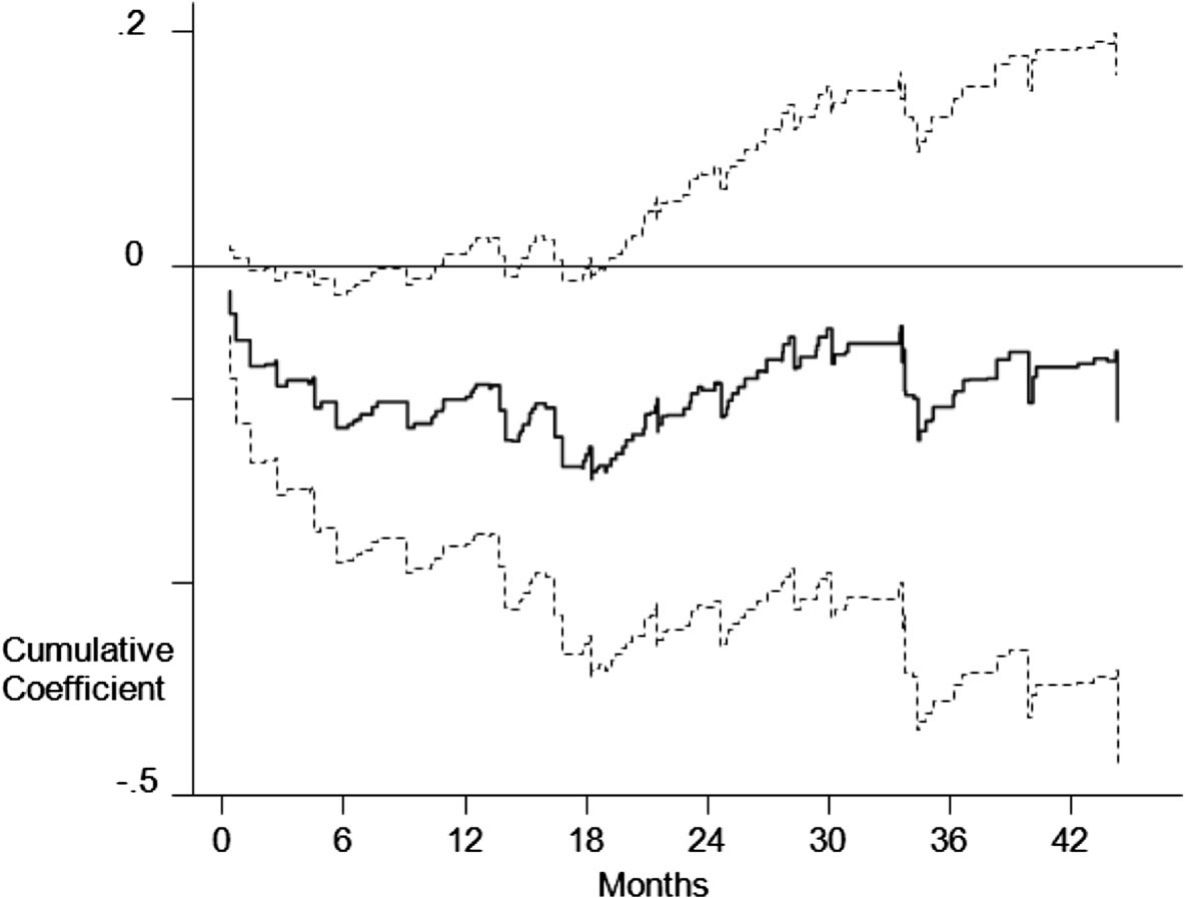
Plot of the cumulative regression coefficient (95 % CI) for the consolidation phase as a function of follow-up time. Aalen’s additive hazard model of progression-free survival from last autologous stem cell transplantation

The plot of the cumulative regression coefficient for the last covariate (consolidation phase) over time showed a decreasing pattern up to the 18^th^ month and then a roughly zero slope. The upper confidence band excluded the null value in the first 18 months (Fig. 3). This plot suggests that the consolidation phase may have had an effect on early follow-up times up to 18 months, but no late effect. The consolidation phase appears to have lost its efficacy after one and half years.

### Weighted Cox proportional hazard models with time-dependent covariate

We performed a weighted Cox proportional hazard model analysis including the consolidation phase as covariate where all measured confounding factors were controlled by weighting. The consolidation phase was specified as an interaction term with the follow-up time. In particular, we specified 2 dummies to denote early and late benefit, one for the first 18 months and the other for the period >18 months, respectively. These dummies were set to zero for patients who did not receive the consolidation phase treatment. The first dummy (early effect of consolidation) was set at 1 for patients who received the consolidation treatment and follow-up time from t_0_ to t_18_ (or t_end of fup_, if previous) and was set to 0 after t_18_. The second dummy (late consolidation effect) was set at 1 for patients who received consolidation treatment and follow-up time from t_18_ to t_end of fup_, and was set to 0 before t_18_.

Consolidation therapy resulted in an early effect with weighted HR = 0.40 (robust 95 % confidence interval 0.17–0.96; *p* = 0.040), whereas it did not show any impact at later follow-up times (weighted HR = 1.98; robust 95 % confidence interval 0.93–4.20; *p* = 0.074). The proportional hazard assumption was satisfied (*p* = 0.9062).

## Discussion

The novelty of this article was to use a propensity score-based approach in an RCT context designed for another purpose to evaluate the effect of the last phase of a complex therapeutic strategy, adjusting for potential selection bias caused by different compliance to the previous phases of the therapeutic scheme. Differential compliance to earlier therapeutic phases of the protocol and the consequent failure of the randomization balance may have caused biased results.

Over the last few decades in different research settings, when the randomization procedure was absent by definition (e.g. observational studies) or was simply lost (e.g. RCTs with low compliance rate), propensity score-based approaches were proposed and became popular. The aim was to re-create an artificial population in which treatment assignment could be ignored, and to compare outcomes in treated and untreated subjects, mimicking randomization when it was absent or when the RCT was designed with another purpose.

Using combined stabilized inverse probability treatment weights, we estimated that consolidation therapy in newly diagnosed multiple myeloma patients had an effect on PFS that was restricted to the first 18 months after the start of therapy.

We applied two sets of weights derived from two distinct PS equations to try to restore balance to receiving autologous stem cell transplantation (ASCT) and to ensure ignorability of the last treatment phase (the so-called consolidation phase). We introduced the PS into the model by inverse probability weighting (IPTW). Stabilization of weights was sufficient to gain robustness.

We used the same approach (IPTW) to calculate weighted Kaplan-Meier survival curves. Adjusted survival curves are very useful in exploratory and descriptive phases and comparing them to unweighted curves permits a simple inspection of the selection forces in a study. In particular, considering survival curves for patients receiving or not receiving the consolidation phase, a selection of poor prognosis patients was evident in the first follow-up period, after which weighted and unweighted curves become similar. The weighted analysis of the consolidation phase showed a time-dependent benefit which was evident for a short time span after ASCT. Subsequently, any advantage disappeared. There was no evidence of interaction between consolidation and randomization arms and the sample size was too small for subgroup analysis.

In a setting such as that of our case study, where the proportional hazards assumption was violated, robustness of weighting procedures is critical. When the effects are time-varying, the gap between survival curves may depend on few risk sets and the weights attributed to a small number of subjects in those risk sets becomes very influential. An exploratory and descriptive solution is to investigate the presence of the time-varying effect through Aalen’s additive hazards models. A weighted version of this model was previously evaluated by Huffer and McKeague [24]. We included time-varying variables in the weighted Cox models as a set of dichotomous covariates after defining time intervals of different lengths based on Aalen’s analysis, so that proportional hazard assumption be satisfied.

## Conclusion

Estimating stabilized inverse probability weights by PS logistic models, combining them to consider the sequence of therapeutic phases, carrying out sensitivity analysis to evaluate the proportional hazard assumption and time-varying effects, fitting Aalen’s additive hazard models to investigate time-varying effects and, finally, estimating a weighted Cox proportional hazard model with time-varying covariates undoubtedly appears a complex strategy. However, it is a more appropriate one to use in the event of lack of compliance to a series of treatment phases and when there is a time-varying effect. Both situations are common in cancer trials.

Notably, PS methods can adjust for imbalance caused by observable covariates. Unobservable covariates can still exert an effect and distort estimates. Thus, analysis using PS methods cannot be conclusive in this context. Ad hoc designed RCTs with randomization procedures planned after ASCT are important to understand what kind of patient really benefits from consolidation treatment.

## Abbreviations

ASCT: Autologous stem cell transplantation
CI: Confidence interval
CR: Complete response
HR: Hazard ratio
IPTW: Inverse probability treatment weight
IQR: Interquartile range
ITT: Intention to treat
MM: Multiple myeloma
Mos: Months
PD: Progression disease
PFS: Progression free survival
PH: Proportional hazard
PP: Per-protocol
PS: Propensity score
RCT: Randomized clinical trial
SIPTW: Stabilized inverse probability treatment weight
TSIPTW: Total stabilized inverse probability treatment weight
